# Effects of oral white willow bark (*Salix alba*) and intravenous flunixin meglumine on prostaglandin E_2_ in healthy dairy calves

**DOI:** 10.3168/jdsc.2021-0138

**Published:** 2021-10-22

**Authors:** H.N. Phillips, K.T. Sharpe, M.I. Endres, B.J. Heins

**Affiliations:** 1 Department of Animal Science, University of Minnesota, St. Paul 55108; 2 West Central Research and Outreach Center, Morris, MN 56267

## Abstract

White willow bark (WWB) is commonly used in combination with other medicinal herbs and analgesics to alleviate inflammatory pain in disbudded calves under organic management, but there is no evidence to confirm an effect of WWB on inflammatory biomarkers in calves. The objective of this study was to determine whether WWB affects the inflammatory biomarker prostaglandin E_2_ (PGE_2_) in healthy dairy calves. A randomized crossover trial with 2 periods and 5 treatments was used for this experiment. A 7-d washout period was used to minimize carryover effects. The treatments were (1) 57.6 mg/kg oral WWB (low dose; L-WWB), (2) 115.1 mg/kg oral WWB (medium dose; M-WWB), (3) 230.3 mg/kg oral WWB (high dose; H-WWB), (4) 2.2 mg/kg i.v. flunixin meglumine (FM), or (5) no treatment (NT). Calves (n = 25) were randomly assigned to receive 1 of the 25 treatment sequences. Blood samples were collected at 1, 2, and 4 h after administration to determine PGE_2_ and salicylic acid plasma concentrations. The WWB had 2,171 μg/g (± 4.3% relative standard error) salicin (0.22%). On average, calves in the FM (721 ± 274 pg/mL) treatment had lower PGE_2_ than calves in all other treatments. Calves in the NT (2,606 ± 271 pg/mL), L-WWB (2,509 ± 276 pg/mL), M-WWB (2,343 ± 270 pg/mL), and H-WWB (3,039 ± 270 pg/mL) treatments had similar PGE_2_ averaged across sampling times. Calves in the L-WWB (23.4 ± 1.9 ng/mL), M-WWB (21.5 ± 1.9 ng/mL), and H-WWB (23.3 ± 1.9 ng/mL) treatments had similar maximum salicylic acid plasma concentrations. Results from this study indicate that the WWB doses used in this experiment were ineffective at achieving dose-dependent PGE_2_ and salicylic acid plasma concentration responses.

## Highlights

• Nonstandardized products with white willow bark had a minute amount of salicin.
• Flunixin meglumine lowered the level of inflammatory biomarker.
• White willow bark did not affect the level of inflammatory biomarker.
• White willow bark did not achieve the salicylic acid concentration needed for analgesia.

## Graphical Abstract

**Figure:**
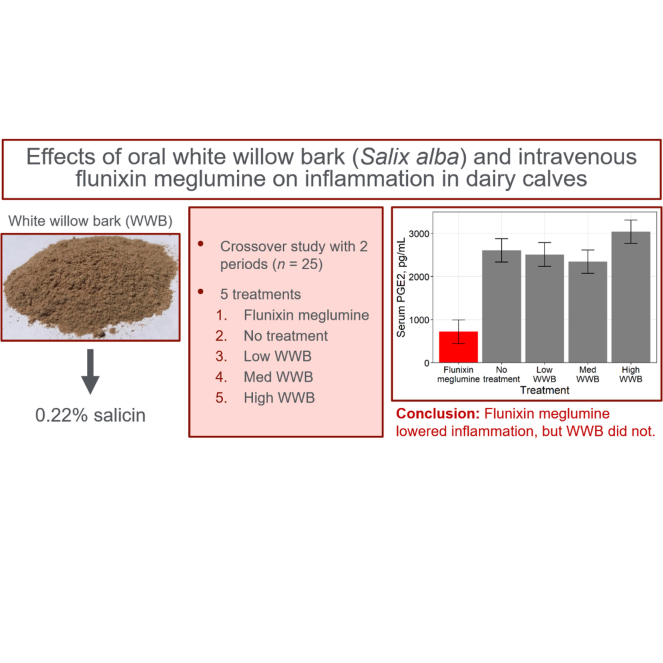
**Summary:** White willow bark is a useful analgesic in humans, and its utility to alleviate pain in organic calves remains of interest. The objectives of this study were to (1) determine the salicin concentrations of non-standardized white willow bark products, and (2) to investigate the effects of intravenous flunixin meglumine and 3 oral doses (low, medium, and high) of white willow bark on the blood plasma concentrations of the inflammatory biomarker prostaglandin E2 and salicylic acid in healthy calves. The white willow bark product had 2,171 μg/g (0.22%) salicin. Flunixin meglumine lowered prostaglandin E2 (PGE_2_), whereas the white willow bark doses were ineffective at reducing PGE_2_ and achieving a minimum plasma salicylic acid concentration necessary for analgesia in calves. Results indicate that the white willow bark doses used in this experiment are unsuitable for producing analgesia in calves.

Dairy calves commonly experience painful disbudding procedures as part of the standard of care. According to Urie et al. (2018), approximately half (52%) of preweaning dairy calves are disbudded, but only 28% of disbudded calves are given pain mitigation therapies for the procedure. Furthermore, a survey of 189 organic dairies in the United States indicated that only 26% use a local analgesic, nonsteroidal anti-inflammatory drug (**NSAID**), or sedation to relieve pain related to horn removal procedures (Bergman et al., 2014). Organic-approved options for pain management are limited to substances approved by the USDA National Organic Program, such as flunixin meglumine (Code of Federal Regulations, 2021). However, even those permitted by the National Organic Program face barriers to common use, such as opposition by farmers, difficulty in administering, and a lack of Food and Drug Administration (**FDA**) approval for use in cattle. Despite this reluctance to implement pain alleviation methods, some organic farmers have expressed interest in or currently implement plant-based alternatives (Pol and Ruegg, 2007; Bergman et al., 2014).

An herbal tincture (Dull It, Dr. Paul's Lab) composed of ethanol, apple cider vinegar, white willow (*Salix alba*) bark, St. John's wort (*Hypericum perforatum*), chamomile (*Matricaria recutita*), arnica (*Arnica montana*), and fennel (*Foeniculum vulgare*) is currently used by many organic dairy producers as a therapy to mitigate disbudding pain and stress. However, the use of this tincture as a drug has not been approved by the FDA and is therefore is not approved for use. The herbal tincture was recently investigated as a therapy for modulating acute cautery disbudding pain in calves, in which the results indicated that the herbal tincture did not reduce the cortisol response but did reduce the behavioral response after disbudding compared with a lidocaine cornual nerve block (Phillips and Heins, 2021). To determine the possible mechanisms underlying the effect of this herbal tincture and other herbal therapies, single constituents of plants and their mechanisms should be investigated further.

Historically, white willow bark (**WWB**) has been used as an anti-inflammatory and analgesic, dating back to ancient civilizations (Maroon et al., 2010). Today, WWB is commonly used to treat painful conditions in humans (Chrubasik et al., 2000; Schmid et al., 2001b; Uehleke et al., 2013). As with all plants in the *Salix* genus, WWB contains salicylate compounds primarily composed of salicin (Kammerer et al., 2005), which is converted to salicylic acid (**SA**) in the body when consumed orally (Mahdi, 2014). Salicylic acid has anti-inflammatory effects similar to synthetic salicylates, such as acetylsalicylic acid (i.e., aspirin) and sodium salicylate, in that it inhibits cyclooxygenases and prevents the formation of prostaglandins and reduces inflammation (Amann and Peskar, 2002; Drummond et al., 2013). However, the authors are not aware of any peer-reviewed published studies indicating the usefulness of WWB for alleviating disbudding pain in calves.

Synthetic salicylates, such as aspirin and sodium salicylate, have historically been used as anti-inflammatories, antipyretics, and analgesics in cattle. Sodium salicylate administered i.v. at 50 mg/kg reduced cortisol concentrations compared with no treatment in cattle following castration (Coetzee et al., 2007). However, aspirin administered orally at 50 mg/kg did not attenuate cortisol (Coetzee et al., 2007). Sodium salicylate dissolved in ad libitum drinking water at rates of 2.5 to 5.0 mg/mL 1 d before and 2 d after castration and dehorning was associated with improved ADG for 13 d and decreased cortisol concentrations for up to 6 h following the procedures compared with calves that received no treatment (Baldridge et al., 2011). Yet despite the historical use of salicylates with cattle, they have never been formally approved by the FDA. Furthermore, unapproved products are currently marketed as if they are approved by the FDA and have undergone clinical research. In general, the leaves and bark of *Salix* spp. are considered safe for livestock consumption (Masika and Afolayan, 2003; Moore et al., 2003). However, the effectiveness of WWB as a pain mitigation method in dairy calves is currently lacking scientific support. Therefore, the objectives of this study were (1) to determine the salicin concentration of nonstandardized products containing WWB that are currently used or may be used for disbudding pain, and (2) to determine the effects of i.v. flunixin meglumine and 3 oral doses of WWB on the inflammatory biomarker prostaglandin E_2_ (**PGE_2_**) and salicylic acid plasma concentrations in healthy calves. The hypotheses of this study were (1) that PGE_2_ plasma concentrations would differ among calves given flunixin meglumine, no treatment, and low, medium, and high doses of WWB; and (2) that maximum salicylic acid plasma concentrations would differ among calves given low, medium, and high doses of WWB.

The salicin concentrations were determined in 3 products: (1) the aforementioned herbal tincture (Dull It, Dr. Paul's Lab), (2) an ethanol-based WWB tincture (Mountain Rose Herbs), and (3) a dried WWB powder (Mountain Rose Herbs). Samples of each product were obtained from a single lot. Samples of the products were analyzed by a commercial laboratory (Eurofins EAG Materials Science, Maryland Heights, MO). Samples were prepared in duplicate and analyzed by HPLC in duplicate; therefore, 4 replicates per sample were analyzed. For sample preparation, the tinctures were diluted in 50% aqueous methanol and passed through a 0.45-μm filter, whereas the powder was suspended in 50% aqueous methanol, sonicated for 10 min, centrifuged at 1,510 × *g* for 15 min, and passed through a 0.45-μm filter. Samples were analyzed by HPLC equipped with a Zorbax SB-C18 phase column (5-μm particle size, 4.6 mm i.d. × 250 mm; Agilent) maintained at 35°C. The injection volume was set to 5 μL, and separation was performed at a flow rate of 1.0 mL/min starting with a solvent composition of 5% acetonitrile, increasing linearly to 20% over 13 min. The solvent composition was increased to 80% acetonitrile over 3 min and held at 80% for 5 min before equilibrating to 5%. Salicin was detected at excitation and emission wavelengths of 210 and 268 nm, respectively. Salicin had a retention time of 8.43 min with a peak retention time of 0.1858% relative standard deviation (**RSD**) and peak area of 1.0041% RSD. For quantitation, a 5-point calibration curve ranging between 10.44 and 100.13 μg/g was generated and had a R^2^ greater than 0.99. The limit of detection was 10.44 μg/g. Quality control samples for the herbal tincture, WWB tincture, and WWB powder had salicin recoveries of 109, 101, and 93%, respectively. The average salicin concentration was greatest for the WWB powder (2,171.2 μg/g ± 4.3% RSD) compared with the herbal tincture (17.6 μg/g ± 3.2% RSD) and the WWB tincture (143.3 μg/g ± 5.0% RSD). Therefore, the WWB powder was used for objective 2.

The experiment for objective 2 was conducted at the University of Minnesota West Central Research and Outreach Center (Morris, MN) during December 2020 using 25 preweaning male calves. All procedures involving animals were approved by the University of Minnesota Institutional Animal Care and Use Committee (# 2007–38250A). Calves were either a crossbreed composed of Viking Red, Montbéliarde, and Holstein or a crossbreed composed of Jersey, Normande, and Viking Red. Calves were (mean ± SD) 56 ± 15 d of age and weighed 85.7 ± 20.7 kg upon study initiation. Calves were housed in a pen consisting of an indoor straw-bedded area (12.2 × 4.9 m) and an outdoor gravel area (10.7 × 4.9 m). Calves were fed pasteurized whole milk from an automated feeding system (CalfExpert Calf Feeder, Holm & Laue GmbH & Co KG). Calves had an 8-L daily allotment of milk in 2.4-L increments. Calves had ad libitum access to water and calf starter (18% CP).

A randomized crossover trial with 2 periods and 5 treatments was used for this experiment. A 7-d washout period was used to minimize carryover effects. The treatments were (1) low dose of WWB (**L-WWB**), (2) medium dose of WWB (**M-WWB**), (3) high dose of WWB (**H-WWB**), (4) flunixin meglumine (**FM**), or (5) no treatment (**NT**). The 25 calves (i.e., experimental units) were randomly assigned to receive 1 of the 25 treatment sequences. The treatment scheme is displayed in Table 1. Calves in the L-WWB, M-WWB, and H-WWB treatments received either 57.6, 115.1, or 230.3 mg of WWB powder/kg orally in boluses, corresponding to 0.125, 0.250, and 0.500 mg/kg salicin, respectively. The WWB treatments were formulated based on the salicin concentration found in the WWB powder as previously described, such that the maximum number of boluses (size 12el, 7.5-mL capacity; Torpac) administered was 5. There are no known studies that use WWB in calves. Therefore, these doses were formulated based on what was presumed to be feasible to give a calf; high doses that require numerous boluses may not be feasible for farmers based on limitations related to cost and labor. The authors agreed before the experiment that investigating doses that represent what farmers might give to their calves would be of most interest. Furthermore, this is the first experiment to investigate WWB in calves; therefore, we found it necessary to err on the side of caution to avoid giving calves potentially large and unforeseen harmful doses. The FM treatment served as the positive control for this study because FM is the only FDA- and organic-approved synthetic NSAID and it has known effects on PGE_2_ concentrations in calves (Fraccaro et al., 2013). Treatment sequences were balanced, and the order of treatment was random. Calves were acclimated to handling 7 d before the study. On study days, treatment administration was staggered by 5 min. Calves in the FM group received 2.2 mg/kg i.v. flunixin meglumine (Banamine, Merck Animal Health). Calves in the NT group received no treatment. Handlers involved in collecting and processing blood samples from calves were blinded to treatments.

**Table 1:** Delineation of calves (n = 25) enrolled in a randomized crossover trial with 2 periods and 7-d washout by treatment sequence^1^

Calf	Period 1	Period 2
1	FM	FM
2	FM	NT
3	FM	L-WWB
4	FM	M-WWB
5	FM	H-WWB
6	NT	FM
7	NT	NT
8	NT	L-WWB
9	NT	M-WWB
10	NT	H-WWB
11	L-WWB	FM
12	L-WWB	NT
13	L-WWB	L-WWB
14	L-WWB	M-WWB
15	L-WWB	H-WWB
16	M-WWB	FM
17	M-WWB	NT
18	M-WWB	L-WWB
19	M-WWB	M-WWB
20	M-WWB	H-WWB
21	H-WWB	FM
22	H-WWB	NT
23	H-WWB	L-WWB
24	H-WWB	M-WWB
25	H-WWB	H-WWB

1 Treatments: FM = 2.2 mg/kg i.v. flunixin meglumine; NT = no treatment; L-WWB, M-WWB, and H-WWB = 57.6, 115.1, and 230.3 mg/kg oral white willow bark in the low-, medium-, and high-dose groups, respectively.

Blood was collected immediately before and 1, 2, and 4 h after treatment via jugular venipuncture (21-gauge × 32-mm; Vacutainer Eclipse, Becton, Dickinson and Co.). Collection times represented the periods of expected maximum SA serum concentration (1 and 2 h) and half-life (4 h) (Schmid et al., 2001a). During each sampling, blood (4 mL per tube) was collected in a sodium heparin tube (Becton, Dickinson and Co.) for PGE_2_ and in a K_2_ EDTA tube (Becton, Dickinson and Co.) for SA determination. Tubes were gently inverted 8 to 10 times, immediately stored in a cooler on ice, and processed within 30 min of collection.

Sample processing for PGE_2_ was as described by Allen et al. (2013). Whole blood (2 mL) was transferred from the collection tube to a 2-mL centrifuge tube (Fisher Scientific) containing 20 µL of 1 mg of LPS (Sigma-Aldrich) per 1 mL of PBS (Alfa Aesar). The centrifuge tube was inverted 3 to 5 times and incubated for 24 h in a 37°C water bath (Isotemp GPD 05, Fisher Scientific). After incubation, tubes were centrifuged (HWLAB 1–12K mini multi speed desktop centrifuge, Fristaden Lab) at 400 × *g* for 10 min before plasma was transferred to cryovials (Fisher Scientific) and frozen at −80°C. For the SA sample processing, blood was centrifuged for 15 min at 10,640 × *g* for 10 min in a chilled centrifuge (4°C), and plasma was transferred to cryovials and frozen at −80°C. Upon study completion, plasma samples were shipped overnight on dry ice to Analytical Chemistry Services (Iowa State University, Ames) for analysis.

For PGE_2_ determination, protein was precipitated from samples in preparation for competitive ELISA (Cayman Chemical). In short, plasma (93.75 µL) with 375 µL of HPLC-grade methanol was centrifuged at 430 × *g*, and the supernatant was decanted into a 5-mL glass culture tube. The solvent was evaporated under a flow of nitrogen in a TurboVap LV (Biotage) at room temperature. The dried extract was resuspended in 375 µL of buffer to a dilution of 1:5. Samples were further diluted to 1:20 with buffer before analysis. Samples were analyzed in duplicate with an 8-point standard curve according to kit instructions. The assay had a detection range of 7.8 to 1,000 pg/mL. Samples were reanalyzed if the coefficient of variation was greater than 20% or if the value was not on the standard curve. Quality control samples were not run with this study, so intra-assay variability was not determined. The inter-assay variability was 10.0%. All curves were linear and had an average R^2^ value of 0.99. Percent binding was 51% over all assays, and nonspecific binding was 0.29%. The limit of detection was 7.8 pg/mL, and the limit of quantitation was 9.60 pg/mL.

Salicylic acid concentration was determined using methods similar to those described by Mathurkar et al. (2018). Salicylic acid concentration was determined using ultra HPLC (Thermo Vanquish Flex, Fisher Scientific) consisting of a binary pump, autosampler, column compartment, variable wavelength UV detector, and a variable wavelength fluorescence detector. Plasma (0.2 mL) was aliquoted for extraction of calibrators, quality controls, and samples. Calibrators were spiked into a blank matrix at 8 concentrations ranging from 20 to 5,000 ng/mL. Three quality control samples were spiked into blank matrices at 150, 1,500, and 3,500 ng/mL. A volume of 20 µL of 12% formic acid was added to each extraction tube, followed by 2 mL of methyl *tert*-butyl ether. Tubes were placed on a multi-tube vortex mixer for 10 min followed by centrifugation at 2,020 × *g* for 5 min at 4°C. The upper layer (1 mL) was transferred and concentrated to dryness at 25°C. Samples were reconstituted in 0.1% aqueous formic acid. The mobile phases consisted of (1) 3.5 m*M* phosphate solution with 0.1% aqueous formic acid, and (2) acetonitrile. Separation was accomplished using an aQ Accucore column (2.6-µm particle size, 2.1 mm i.d. × 100 mm; Fisher Scientific) maintained at 45°C. The autosampler was maintained at 6°C and the injection volume was set to 5 µL. The separation was performed at a flow rate of 0.3 mL/min at a starting solvent composition of 25% acetonitrile, increasing linearly to 35% acetonitrile over 3.5 min. The solvent composition was then increased to 95% acetonitrile over 0.5 min and held at 95% acetonitrile for 2 min before equilibrating to 25% acetonitrile. Salicylic acid was detected at an excitation wavelength of 295 nm and an emission wavelength of 410 nm and had a retention time of 1.92 (SD = 0.019) min. Thermo Chromeleon software (Fisher Scientific) was used to process quantitative results. A calibration consisting of 8 points between 20 and 5,000 ng/mL and a blank resulted in a linear curve with an R^2^ of 0.99. The lower limit of quantification was 20 ng/mL. All quality control samples were calculated within 20% of their nominal value.

All statistical analyses used the 1.4.1103 version of the RStudio software (https://www.rstudio.com/). Analyses were performed using the *lmer* function of the *lme4* package (Bates et al., 2015). The *lmer* function fits a linear mixed-effects model. For the analysis of PGE_2_, the model included fixed effects for baseline PGE_2_ (continuous), BW (continuous), period (2 levels), time (3 levels), treatment (5 levels), and time × treatment interaction, and random intercepts for calf (25 levels) and calf within period to account for repeated measures (i.e., correlations between subjects). Baseline PGE_2_ was analyzed in a separate model with fixed effects of BW, period, and treatment, and a random intercept for calf. For the analysis of SA, the NT and FM treatments were removed, and the maximum SA value was identified for each calf. The model for maximum SA included fixed effects for period and treatment and a random intercept for calf. Order of treatment (continuous) and breed (2 levels) were considered fixed effects candidates but were excluded from the models based on lack of improved model fit. Continuous predictors were centered and scaled to have a mean of 0 and SD of 1 for all models. The REML parameter estimates were used to calculate the LSM and SEM, and *F*-tests were used to evaluate the significance of main effects. The Kenward-Roger approximation was used to calculate denominator degrees of freedom. The Tukey adjustment was applied to compare treatment means if the corresponding main effect had *P* < 0.05.

Similar baseline PGE_2_ values (LSM ± SEM) were observed for calves in the FM (2,443 ± 442 pg/mL), NT (2,846 ± 444 pg/mL), L-WWB (3,170 ± 443 pg/mL), M-WWB (2,825 ± 443 pg/mL), and H-WWB (2,800 ± 441 pg/mL) treatments (*F*_4,43_ = 0.3, *P* = 0.85).

For the analysis of post-treatment PGE_2_, we detected no interaction between time and treatment (*F*_8,90_ = 2.0, *P* = 0.05). However, there were effects of time (*F*_2,90_ = 3.8, *P* = 0.03) and treatment (*F*_4,37_ = 11.5, *P* < 0.01). When averaged across all post-treatment time points, calves in the FM group had lower PGE_2_ compared with calves in all other treatments (Table 2). The concentration of PGE_2_ (LSM ± SEM) was greater (*P* = 0.03) at 2 h (2,396 ± 164 pg/mL) than at 4 h (2,010 ± 164 pg/mL), whereas PGE_2_ concentration at 1 h (2,324 ± 164 pg/mL) was intermediate compared with that at the other time points (*P* ≥ 0.09).

**Table 2:** Least squares means Â± SEM for the effect of treatment on plasma concentrations of prostaglandin E_2_ (PGE_2_) and maximum salicylic acid (SA) in calves (n = 25) enrolled in a randomized crossover trial with 2 periods and 7-d washout^1^

Plasma concentration	Treatment^2^				
	FM	NT	L-WWB	M-WWB	H-WWB
PGE_2_, pg/mL	721 Â± 274^b^	2,606 Â± 271^a^	2,509 Â± 276^a^	2,343 Â± 270^a^	3,039 Â± 270^a^
Maximum SA, ng/mL	—	—	23.4 Â± 1.9	21.5 Â± 1.9	23.3 Â± 1.9

a,b Means within a row with different letters are different at *P* < 0.01.

1 Blood samples for determining plasma concentrations of PGE_2_ and maximum SA were taken at 1, 2, and 4 h after treatment administrations.

2 Treatments: FM = 2.2 mg/kg i.v. flunixin meglumine; NT = no treatment; L-WWB, M-WWB, and H-WWB = 57.6, 115.1, and 230.3 mg/kg oral white willow bark in the low-, medium-, and high-dose groups, respectively.

The L-WWB, M-WWB, and H-WWB treatments had similar maximum SA (Table 2; *F*_2,7_ = 1.2, *P* = 0.36). Only 5 calves that received the WWB treatment achieved an SA plasma concentration greater than the lower limit of quantification (20 ng/mL); 4 received the H-WWB treatment and 1 received the L-WWB treatment. Maximum SA concentrations were only observed at 1 h (3 calves) and 2 h (2 calves).

This research is the first to report the use of WWB in calves. The WWB product used in this study had 2,171 μg/g (0.22%) salicin. However, the concentration of salicin may vary between lots. As expected, the FM treatment successfully reduced inflammatory mediators in calves, as indicated by lower PGE_2_ values, compared with the NT treatment. However, none of the 3 doses of WWB reduced PGE_2_, and the maximum SA plasma concentrations were similar among the L-WWB, M-WWB, and H-WWB treatments, indicating that the treatment doses might have been too low. Furthermore, most calves that received the WWB treatments had undetectable SA plasma concentrations, indicating that the doses of WWB were too low to detect.

Salicin is the most notable medicinal compound in WWB extracts. After ingestion, salicin is converted to metabolites in the salicylate family, which can be detected in the plasma of blood. There are several compounds that are considered salicylates, but SA is the major metabolite that makes up total salicylates detected in the plasma after ingesting salicin. In a pharmacokinetics experiment of oral WWB in humans, salicylic acid was the major metabolite (86% of total salicylates) of salicin detected in the serum (Schmid et al., 2001a). In Schmid et al. (2001a), humans with an average BW of 69.4 kg consumed a total of 1,360 mg of standardized WWB extract (240 mg of salicin) over 2 time points 3 h apart. The maximum SA plasma concentration (8.4 µmol/L) was reached after the second dose at 4 h, which was equal to 1.16 µg/mL, given the molar mass of SA (0.0084 µmol/mL × 138.121 g/mol = 1.16 µg/mL).

There are very few studies on the pharmacokinetics and pharmacodynamics of salicin. However, similar compounds, such as aspirin and sodium salicylate, also form salicylate metabolites and have been studied more intensively. The minimum total salicylate plasma concentration needed for analgesia in calves was previously estimated to be 25 to 30 μg/mL (Gingerich et al., 1975; Coetzee et al., 2007). Because SA makes up an estimated 86% of total salicylates in the plasma after consumption of salicin (Schmid et al., 2001a), the estimated minimum SA plasma concentration needed for analgesia in calves is approximately 21.5 to 25.8 μg/mL.

Previous studies of aspirin and sodium salicylate administered orally in ruminants suggest that greater doses than those used in the present experiment are needed, coupled with more frequent administration. For example, single doses of aspirin in calves (50 mg/kg) and sodium salicylate in sheep (200 mg/kg) both failed to achieve plasma salicylate concentrations above 10 μg/mL (Coetzee et al., 2007; Mathurkar et al., 2018), but aspirin at 100 mg/kg every 12 h maintained plasma salicylate concentrations above 30 μg/mL in dairy cows (Gingerich et al., 1975). Similarly, 2 daily aspirin doses of 200 mg/kg over the first 2 DIM reduced clinical metritis at 7 and 14 DIM (Barragan et al., 2021), and 3 daily sodium salicylate doses of 185 mg/kg over the first 3 DIM increased early-lactation milk yield (Carpenter et al., 2016).

The area under the curve of SA plasma concentration obtained in Schmid et al. (2001a) after humans consumed WWB extract corresponding to 240 mg of salicin (13.67 μg·h/mL) was similar to that expected after a single aspirin dose of 80 mg (12.60 μg·h/mL) and 100 mg (14.6 μg·h/mL) in humans (Benedek et al., 1995; Nagelschmitz et al., 2014). Therefore, the estimated dose of salicin can be estimated by multiplying the aspirin dose by a factor of 2.6 to 2.8. Furthermore, aspirin doses of 100, 300, and 500 mg in humans had a linearly proportional relationship with the area under the curve and maximum concentration for plasma SA (Nagelschmitz et al., 2014). Mathurkar et al. (2018) compared 2 oral doses of sodium salicylate in sheep and reported that 100 and 200 mg/kg yielded maximum SA plasma concentration values of 4.22 and 8.27 μg/mL, respectively. Based on the previous information and a linearly proportional relationship between dose and maximum concentration, calves would need sodium salicylate at a dose of approximately 520 mg/kg to reach the minimum SA plasma concentration for analgesia in calves (21.5 μg/mL). Alternatively, a total aspirin dose of 400 mg/kg given over the course of several time points may also be adequate for reducing inflammatory biomarkers (Barragan et al., 2021). After multiplying these doses by a factor of 2.6 to 2.8, the estimated dose range of salicin needed for analgesia in calves is 1,040 to 1,456 mg/kg. The dose could be given over several time points to prevent gastrointestinal upset and stress to the calves. Previous studies use maximum single aspirin and sodium salicylate doses of 200 mg/kg (Mathurkar et al., 2018; Barragan et al., 2021), so salicin doses greater than 200 mg/kg at a single time point should be administered with precaution.

The estimated amount of salicin needed to achieve analgesia in calves is quite large, considering that WWB has a minute amount of salicin. Even if a standardized WWB extract, such as a 15% salicin product, were used, it would have to be given at a total dose of approximately 6,933 to 9,707 mg/kg (equivalent to 1,040 to 1,456 mg/kg of salicin). This dose could be given over 1 to 3 d in drinking water or milk, as demonstrated with aspirin and sodium salicylate in other studies (Carpenter et al., 2016; Barragan et al., 2021). However, this method may be impractical considering time and financial constraints. Furthermore, there is currently no evidentiary support for whether WWB at high doses given over several days has any effect on inflammatory biomarkers in calves. Furthermore, other constituents of WWB might be toxic and have unknown pharmacokinetics and therefore withdrawal times. In fact, sustained high doses of WWB may have negative effects on health and welfare, such as gastrointestinal upset and consequent increased inflammation, as demonstrated in adult cattle given aspirin orally (Briggs et al., 2020).

In conclusion, the results of the current experiment reveal that products containing nonstandardized WWB have a very small amount of salicin, and the necessary dose of WWB to reduce inflammatory biomarkers and achieve a SA plasma concentration required for analgesia in calves was not determined. In fact, the WWB doses evaluated in the present experiment were likely much lower than what would be required for an appropriate dose-dependent response. The proper WWB dose for analgesia in calves is untested and may have unforeseen negative effects on animal wellbeing. Further research should focus on finding a dose of WWB or salicin that achieves a SA plasma concentration necessary for analgesia in calves before testing the efficacy of WWB under farm settings.
